# Astrocytoma: A Hormone-Sensitive Tumor?

**DOI:** 10.3390/ijms21239114

**Published:** 2020-11-30

**Authors:** Alex Hirtz, Fabien Rech, Hélène Dubois-Pot-Schneider, Hélène Dumond

**Affiliations:** 1 Université de Lorraine, CNRS, CRAN, F-54000 Nancy, France; alex.hirtz@univ-lorraine.fr (A.H.); fabien.rech@univ-lorraine.fr (F.R.); helene.dubois-pot-schneider@univ-lorraine.fr (H.D.-P.-S.); 2 Université de Lorraine, CHRU-Nancy, Service de Neurochirurgie, F-54000 Nancy, France

**Keywords:** steroids, estrogens, receptor isoforms, signaling, astrocytoma, glioblastoma, gender, pregnancy

## Abstract

Astrocytomas and, in particular, their most severe form, glioblastoma, are the most aggressive primary brain tumors and those with the poorest vital prognosis. Standard treatment only slightly improves patient survival. Therefore, new therapies are needed. Very few risk factors have been clearly identified but many epidemiological studies have reported a higher incidence in men than women with a sex ratio of 1:4. Based on these observations, it has been proposed that the neurosteroids and especially the estrogens found in higher concentrations in women’s brains could, in part, explain this difference. Estrogens can bind to nuclear or membrane receptors and potentially stimulate many different interconnected signaling pathways. The study of these receptors is even more complex since many isoforms are produced from each estrogen receptor encoding gene through alternative promoter usage or splicing, with each of them potentially having a specific role in the cell. The purpose of this review is to discuss recent data supporting the involvement of steroids during gliomagenesis and to focus on the potential neuroprotective role as well as the mechanisms of action of estrogens in gliomas.

## 1. Glioma

### 1.1. Etiology

Gliomas are among the most frequent and aggressive primary tumors of the central nervous system (CNS) in adults. The nomenclature of gliomas depends on the glial cell of origin. Astrocytomas and glioblastomas (GBMs) refer to gliomas of astrocytic origin, oligodendrogliomas of oligodendrocytes and ependymomas of ependymal cells, respectively. In this review, we will focus on adult astrocytomas, oligodendrogliomas and GBMs that represent 90% of glioma cases. The causes of tumor emergence and development are currently unknown, since only a few risk factors have been described. For example, patients who were already exposed to ionizing radiation are twice as likely to develop a glioma. Some genetic syndromes (Cowden, Turcot, Lynch, Li-Fraumeni, Neurofibromatosis type I) are also responsible for increasing the risk of developing a glioma. However, these risk factors are very specific to few cases and do not explain the majority of brain tumors.

### 1.2. Classification

The 2007 WHO Classification of Tumors of the Central Nervous System [1] used morphological and immunohistochemical characteristics to establish the glioma classification (Table 1) [1]. Four grades (I to IV) were described, depending on the severity of the tumor with the highest grade (IV-GBM) corresponding to the highest severity [1]. The grade I tumors or pilocytic astrocytomas are often benign and can be resected by surgery. Grade II are also called diffuse gliomas, infiltrative or low-grade gliomas and include astrocytoma, oligodendroglioma and oligoastrocytoma. Grade II are slow-growing tumors that will acquire a more aggressive phenotype over time. The transition from grade II to grade III is measured by assessing tumor cell density and differentiation, number of atypical nuclei and mitotic index. Grade III are anaplastic astrocytomas, oligodendrogliomas and oligoastrocytomas. Grade IV-GBMs are characterized by a fast-growing phenotype with the presence of necrotic regions and vascular development [2]. Primary GBMs develop de novo whereas secondary GBMs originate from lower grade glioma.

Although this type of classification was useful to diagnose the severity of the disease, it remained often ineffective to drive the treatment decision and predict the patient survival. In 2016, the WHO classification was updated by including some molecular signatures (Table 2) [3]. This provided new elements to refine glioma classification and define new subtypes related to patient outcome. This new classification now includes isocitrate dehydrogenase (IDH) mutation and 1p/19q codeletion [4].

IDH proteins are involved in tricarboxylic acid cycle and catalyze the conversion of α-ketoglutarate into citrate. The most common mutation of the IDH1 gene is the R132H, which triggers a tridimensional change of the enzyme structure. The mutated conformation impacts the enzymatic activity which becomes able to convert α-ketoglutarate to D-2-hydroxyglutarate (D2HG). The accumulation of this oncometabolite is harmful for the cell since it induces epigenetic modifications (histone modification and DNA methylation) resulting in aberrant gene expression [5]. Indeed, D2HG acts as an inhibitor of several α-ketoglutarate-dependent dioxygenases, including histone demethylases or TET 5-methylcytosine hydroxylases, leading to an increase in repressive histone methylation marks and a global increase in 5-methylcytosine level. Interestingly, in glioma, IDH mutations are associated with a more favorable prognosis because these tumors are more sensitive to the chemotherapeutic drug, temozolomide (TMZ) [6].

In addition to IDH mutations, 1p/19q loss of heterozygosity alteration is used for classification of glioma. The chromosomal 1p/19q codeletion (hallmark of oligodendroglioma) associated with IDH mutation increases the range of response to alkylating agent chemotherapy and thus leads to an even better prognosis [7,8].

Other genetic alterations, not required for diagnosis, are currently investigated in clinics to predict the response to treatment: ATRX (alpha thalassemia/mental retardation syndrome X-linked), TERT (telomerase reverse transcriptase), EGFR (epidermal growth factor receptor), BRAF (V-raf murine sarcoma viral oncogene homolog B1), histone H3K27M mutations and MGMT (O-6-methylguanine-DNA methyltransferase) promoter methylation (Table 2) [9]. MGMT is a DNA repair protein involved in DNA damage repair. The methylation of MGMT promoter is linked to a favorable prognosis in glioma treated by TMZ since MGMT expression is decreased, which leads to impaired DNA repair ability in tumor cells and enhances the sensitivity to alkylating agents [10].

A comparison of WHO 2007 and 2016 classifications is presented in Table 1 and Table 2.

### 1.3. Current Treatment

The current treatment for gliomas is a combination of surgery, chemotherapy (mainly TMZ) and radiotherapy that results in patient outcome regarding the subtypes of gliomas (from 18 months of median overall survival in GBM to more than 15 years of median overall survival in 1p19q codeleted oligodendroglioma).

Even if the new 2016 classification is now commonly used for the diagnosis of glioma grades, a few treatments are currently on trial for precision medicine [11]. Large-scale genomic studies identified several frequently mutated genes in glioma, among which IDH1/2, p53, RB and altered receptor tyrosine kinase (RTK) signaling pathways [12]. Specific drugs targeting each pathway, especially the IDH1 or IDH2 inhibitors ivosetinib or enasidenib (clinical trial: NCT02273739), are currently tested as new therapeutic opportunities [13,14].

### 1.4. Evolution of Worldwide Incidence/Survival

A study aimed to compare the worldwide incidence rates of CNS cancers between 1990 and 2016 showed a 17.3% increase worldwide, reaching 32.5% for western Europe [15]. From a cohort of 264,241 pediatric and adult patients affected by various CNS tumors between 2003 and 2007, Leece and colleagues [16] established that the incidence was 5.57/100,000 for overall CNS tumors, and average of 2.98/100,000 for astrocytic tumors, which preferentially affected patients over 45 years of age [16]. The five-year survival was less than 3% for GBM, the highest-grade astrocytoma [17]. For patients over 40, the overall incidence rose up to 11.75/100,000 and 6.77/100,000 for astrocytic tumors [16]. Over 50 years of age, the epidemiological data collected by several teams from different countries indicated that the incidence rate depended mainly on three intrinsic parameters: age (over 40 years), ethnicity and gender [16,18,19,20,21,22]. Leece and colleagues [16] estimated a lower incidence rate of brain and other CNS tumors in all age groups among Asians (male: 4.0–6.5/100,000; female: 2.4–4.3/100,000) compared to Caucasians of all ages (male: 8.2–8.7/100,000; female: 5.3–5.6/100,000) [16]. For people aged over 40 years, the lowest rate was reported for East/Southeast Asia (1.91/100,000) and the highest for Australia and New Zealand (9.58/100,000) followed by the US and western Europe (8.45/100,000). Another worldwide comparative study of the incidence rate for CNS cancers in men and women (aged 0–75 years old) also showed a significant divergence: in particular, in western Europe, incidence rate in 2012 was 6.1/100,000 and 4.4/100,000 for men or women, respectively. Based on Ostrom and colleagues [23], men are 1.58-fold more likely to develop GBMs than women. Likewise, a sex ratio of around 1.30 is verified for oligodendrogliomas and other astrocytomas [23].

Recently, sex differences in the incidence of GBMs have been used to try refining the molecular-based 2016 classification of gliomas [24]. Using a computational algorithm that linked magnetic resonance imaging data, patient outcome data and transcriptomes available in the TCGA database, Yang and colleagues [24] first confirmed that women had longer overall survival than men, whatever the molecular status of GBM [24]. The IDH1 mutation conferred a survival benefit among patients from both sexes. Second, they performed gene clustering to evaluate sex-specific impact of IDH1 mutation on patient outcome. The survival benefit of the female “long survivors” was independent of IDH1 mutations whereas IDH1 status exerted a significant influence among the male “long survivors”, suggesting that being a female (IDH1 wt or mutated) provided a better survival advantage than being an IDH1-mutated male. However, in sex-specific expression profiles associated with male or female “long survivor” clusters, no gene was directly related to hormone synthesis or signaling. Therefore, a more detailed analysis of hormone-response pathways in GBMs as well as in low-grade gliomas is needed to understand the sex-based differences in tumor development and take such data into account to further refine glioma classification.

In conclusion, the main intrinsic risk factors for CNS tumors and especially gliomas of astrocytic origin are age over 40 years, male gender and Caucasian origin, which may combine to currently unknown extrinsic factors such as fetal lifelong exposure to unknown compounds, healthcare, diet and lifestyle.

## 2. Gender Differences in Astrocytoma: The Hypothesis of Steroid Hormones

Epidemiological studies indicate that, whatever the age, ethnicity and socio-economic parameters, the incidence of glioma is higher in men than women with a sex ratio of 1.4:1, raising the question of underlying mechanisms, including the potential role of hormones in the development of these tumors.

In adults, the incidence of GBM is about 40% higher in men than in women. In the United States, the annual age-adjusted incidence rate is 7.6 per 100,000 for men and 5.4 per 100,000 in women, with a stable excess of cases in men, over time. In addition, the ratio for women vs. men begins to decline in pre-menopausal age, reaching 0.51 in people aged 50–54 years [25,26,27,28]. Subsequently, this ratio increases again and remains at a constant level of about 0.65 in parallel with the decrease in hormone levels in women [26]. These results strongly suggest that female hormones have preventive effects on gliomagenesis.

### 2.1. Steroid Biosynthesis

**Figure 1:**
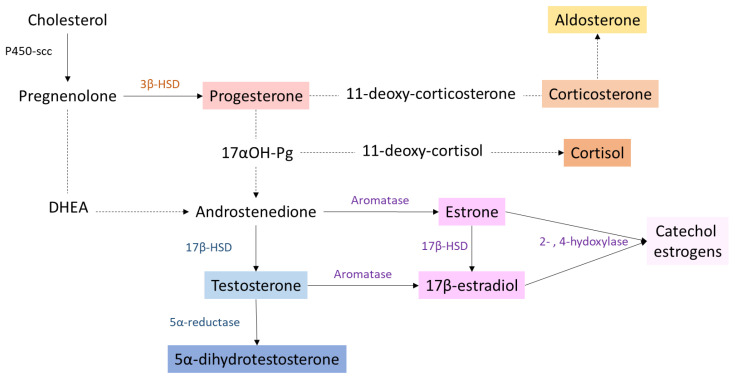
Simplified scheme of steroid biosynthesis pathway. Main enzymes that convert one steroid to another are indicated with plain arrows. Dotted lines represent multiple enzymatic reactions. P450-scc: cholesterol side chain cleavage enzyme; 3β-HSD: 3-β hydroxysteroid dehydrogenase; 17β-HSD: 17-β hydroxysteroid dehydrogenase; DHEA: dehydroepiandrostedione; 17αOH-Pg: 17α-hydroxyprogesterone.

Steroid hormones are synthesized from cholesterol (27 carbons) via an enzymatic biosynthetic pathway that is mainly active in the ovaries and adrenal glands in premenopausal women and in placenta during pregnancy (Figure 1). When ovarian function ceases at the time of menopause, steroids are produced in smaller quantities by peripheral tissues, such as the adrenal glands, liver, brain and fat tissue. In men, steroidogenesis mainly occurs in testes. It is initiated in Leydig cells and ends with the androgen aromatization in the Sertoli cells [29,30]. There are also secondary sources of steroids, produced by brain and peripheral tissues. Immediately after their synthesis, steroids are released into the bloodstream. To allow their transport to the target tissues, these lipophilic hormones bind the SHBG (sex hormone binding globulin). They can also bind in a non-specific way to albumin or remain in free form (for example, 2–3% of circulating estradiol remains unbound).

Steroidogenesis begins with the cleavage of the cholesterol side chain by the cytochrome P450scc (side chain cleavage) enzyme located on the inner mitochondrial membrane. This first step leads to the formation of pregnenolone, which then reaches the endoplasmic reticulum where it will be transformed into progesterone under the action of the enzyme 3β-HSD (3β-hydroxysteroid dehydrogenase). Following the intervention of two oxidoreductases and a lyase (17α-hydroxylase, 17-20 lyase, 17 β-HSD), progesterone is converted to androgens, androstenedione and testosterone, which will be transformed by the cytochrome P450 aromatase into estrogens (oestrone (E1)) and 17β-estradiol (E2)) or converted to 5α-dihydrotestosterone (DHT) by 5α-reductase, respectively. Estriol (E3) is obtained from E1 or E2 through the action of enzymes cytochrome P450 (CYP3A4, CYP3A5) expressed only in the liver and placenta. Finally, estetrol (E4) is synthesized from E2 and E3 in the fetal liver by the action of enzyme of 15α- and 16α-hydroxylases [31]. Then, 17β-estradiol and oestrone can be hydroxylated in the C2, C4 or C16 position by different cytochrome P450 enzymes. This leads to the synthesis of derivatives called catechol estrogens (2-,4- or 16-αhydroxyestrone and 16-αhydroxyestradiol). These compounds can then be metabolized to methoxyestrogens by the action of the enzyme, catechol-O-methyltransferase (COMT). In order to allow their elimination in urine or feces, all of these compounds (estrogens, catechol- and methoxyestrogens) may be conjugated with glucuronic acid or a sulphate group by liver enzymes: UDP-glucuronosyltransferases and sulfotransferases [32].

The glucocorticoids, 11-deoxycortisol and 11-deoxycorticosterone are produced from 17-hydroxyprogesterone (17αOH-Pg) and progesterone, respectively, by the action of 21-hydroxylase (CYP21A2), followed by the P450C11 (cyp11, cytochrome P450 11β-hydroxylase B1 and/or B2) activity.

### 2.2. Steroid Functions in Normal Brain

Even if the main number of steroids is produced in organs distant from the brain (i.e., gonads, adrenal cortex and placenta), they can cross the blood–brain barrier or be produced in situ and exert multiple roles into the CNS [33]. Steroid hormones are involved in numerous brain functions, including the development of the central and peripheral nervous system, the regulation of neurotransmitter metabolism, synaptic connectivity, dendritic branching and myelination [34].

In the human developing brain (from gestational to neonatal period), sex hormones determine apoptosis, neuronal migration, neurogenesis, axonal guidance, synaptogenesis while the estradiol/testosterone balance induces sexual differentiation of the brain, may condition neuronal stem cell survival and differentiation, and thus late onset of neurodegenerative syndromes. At the cerebral level, all the enzymes necessary for estrogen biosynthesis (mainly P450scc, 3β-HSD, 17β-HSD and aromatase) are present in neurons and some populations of astrocytes and oligodendrocytes in the hypothalamus, arcuate nucleus, olfactory bulbs, pre-optic areas and certain regions of the cortex, hippocampus and thalamus [35,36]. Therefore, pregnenolone, progesterone, testosterone and 17β-estradiol can be synthetized de novo in normal brain [37]. Catechol estrogens formation and metabolism also occur in brain tissue, but their role and mode of action remain to be investigated [38].

Estrogens are circulating steroid hormones mainly produced by the gonads through the action of aromatase. Aromatase is also expressed in non-reproductive tissues such as the liver, heart, muscles, bones and brain, highlighting the important role of estrogens in controlling the pathophysiology of many organs and the diversity of their targets. Estrogens regulate glucose transport (GLUT transporter), aerobic glycolysis (hexokinase and pyruvate dehydrogenase activity), and mitochondrial function (aconitase and ATP synthase) to generate ATP. Estrogens also cooperate with adipokines such as leptin and ghrelin to coordinate cerebral control of the whole body’s food intake/energy expenditure and reproductive function [39,40,41]. Estradiol has also been reported to modulate motor control, social and cognitive behavior, mood and pain sensitivity [42]. From mid-pregnancy to birth, progesterone, originating from both maternal and fetal tissues, also participates in myelination, neuroprotection, brain sexual differentiation and neural circuits maturation [43].

How androgens work in the brain will depend on the type of androgen. Dehydroepiandrostenedione (DHEA) is involved in the development of cortico-amygdala and cortico-hippocampal structural networks regulating attention, working memory, reading and writing abilities. Testosterone modulates aggression, cognitive flexibility and may have a positive effect on subventricular zone cell proliferation [44,45]. Testosterone and DHEA seem to have a positive effect on newborn neuron survival [46].

Endogenous glucocorticoids—mainly cortisone and cortisol—are also essential for normal brain development. Normal levels of glucocorticoids are necessary for the proliferation, differentiation and survival of neural stem cells that express glucocorticoid receptors (GRs) in the early stages of fetal development [47]. In humans, glucocorticoids affect excitement, sleep, behavior, cognition, memory, mood and attachment [48]. In the case of stress, increase in blood glucocorticoid levels could impact the cerebral development of the fetus during gestation and promote schizophrenia in adult life [49].

### 2.3. Clinical Data: The Female Hormone Paradox

Several teams showed that the risk of developing GBM is reduced in women who have taken contraceptives or hormone replacement therapy (HRT) containing a mixture of estrogens and progestins suggesting a protective role of the female hormone, whereas other studies reported a deleterious effect of pregnancy on glioma anaplastic progression [50,51,52].

#### 2.3.1. Contraceptive Pills and Hormone Replacement Therapy: The Two Facets of Exogenous Hormones

In order to explain the differential incidence between men and women, many studies addressed the impact of exogenous hormone intake on glioma onset. A recent meta-analysis carried out on five patient cohorts and 11 case-control studies for a total of 8,055,027 women addressed the effect of exogenous hormone use on glioma risk [52]. The age of the patients was very variable among the selected studies preventing an age-related analysis. They compared two types of exogenous hormone intake: the use of oral contraceptives (OC) or HRT. In the case-control studies, they reported an odds ratio (OR) of 0.91 for HRT and 0.99 for OC demonstrating a very low impact of hormones. However, in the cohort studies, there was a relative cancer risk (RR) of 0.95 for HRT and 0.75 for OC showing a potential neuroprotective effect of the hormones. As the estrogen level of women varies throughout life, it would have been also interesting to group female patients according to their menopausal status. Felini and colleagues [51] also reported that post-menopausal women under HRT are less likely to develop a glioma (OR 0.56) [51].

Other studies conducted specifically in post-menopausal women did not report the beneficial effects of OC intake during reproductive age on the onset of glioma after menopause [53]. Although there were several types of contraceptive pills, those based on estrogen or progestin had no impact on the appearance of gliomas [54]. One missing point in these studies is whether the women in the cohort were still on hormonal treatment (HRT) even after menopause.

A case-control study was conducted on pre-menopausal women including 317 cases for 2126 controls in order to study the impact of contraceptive pill intake on the risk of developing glioma. The odds ratio (OR) for progestogen contraceptives alone is 2.8 which is higher than the combination of estrogen and progestogen pills (OR 1.4) [55]. The presence of estrogens in certain types of pill seemed to reduce the negative impact of progestin alone on glioma risk. This study could support the neuroprotective effect of estrogens.

Overall, these studies indicated that progesterone was more likely to cause gliomas and that estradiol might counteract these effects.

#### 2.3.2. Hormone Burst During Pregnancy: A Trigger Toward Disease Progression?

The link between glioma risk, the age of first pregnancy or the number of gestations was also addressed. Two studies from Felini, Forster and colleagues [51,56] reported no correlation between these events and the appearance of gliomas [51,53,56].

Paradoxically, a study from the neuro-oncology department of Nancy hospital, involving 11 pregnant women harboring grade II gliomas (oligodendrogliomas and astrocytomas), showed in 75% cases a significantly increased in tumor growth rate and a 40% increase in seizure frequency [57]. More recently, Peeters and colleagues [58] and Van Westrhenen and colleagues [59] reported that, during pregnancy, tumor growth accelerated (87% of cases) and associated with early clinical deterioration (38% of cases) in gliomas from grade II to IV compared to the general patient population [58,59]. During pregnancy, the level of estrogen and progesterone may increase by 200-fold and is a lead to explain the harmful effect on glioma progression.

Thus, some clinical observations highlighted a potential impact of steroid hormones on gliomagenesis. In order to confirm and validate these findings, numerous in vivo and in vitro studies have been carried out.

### 2.4. Animal and In Vitro Studies

#### 2.4.1. Exogenous Estrogen Exposure: A Promising Track to Modulate Tumor Hallmarks

The impact of 17β-estradiol on tumor progression was addressed on several groups of male, female and ovariectomized female (OVX) rats, orthotopically xenografted with human GBM cells [60]. In the absence of treatment, females had a higher survival (median survival 29 days) than OVX females (median survival 22 days) and males (median survival 23 days). The OVX females once treated with physiological estrogen concentrations (12 µg/day for 21 days) recovered their survival advantage over males. These data suggested that exogenous estradiol might be responsible for the better survival of exposed rats. However, very little information is available on the molecular mechanisms underlying these phenotypes.

A study conducted by Altiok and colleagues [61] in vitro showed that 17β-estradiol inhibited the growth of T98G (human male GBM cells) and C6 rat glioma cells (IC50: 3.8 µM and 3.5 µM, respectively) [61]. A highest concentration of 20 µM promoted cell apoptosis via activation of the JNK-c-jun pathway [61]. The teams of Bishop and Castracani [62,63] described opposite effects since 2 nM or 5 nM of 17β-estradiol added to C6 and U87 (human male GBM cells) culture medium increased cell proliferation [62,63]. Taken together, these conflicting results underscored the involvement of estrogens in the control of glioma cell proliferation depending on the doses and the cell lines used.

Doses ranging from 0.1 µM to 1 nM 17β-estradiol also caused an increase in the migratory and invasive abilities of both human male GBM cells, U87 and T98G. This would be due to a 40–60% 17β-estradiol-dependent decrease in aquaporin 2 (AQP2) mRNA and protein levels [64]. The 17β-estradiol doses ranging from 10 to 100 nM increased the migratory potential of C6 cells while no significant effect was observed for the F98 rat embryonic glioma cell line, demonstrating, once again, the importance of cellular model [65].

Notably, 17β-estradiol can be metabolized by glial cells into 2-methoxyestradiol (2-ME), whose affinity is 2000-fold less than 17β-estradiol for estrogen nuclear receptors but 2-fold higher for the membrane g-protein coupled estrogen receptor, GPER. Concentrations ranging from 2 to 20 µM of 2-ME caused a decrease in cell viability in multiple GBM cell lines, U87, U138 (human male GBM cell line), LN405 (human female GBM cell line) and RG-2 (rat GBM cell line) by more than 75%, associated with an increase in apoptosis [66]. Lis and colleagues [67] also reported a decrease in cell proliferation with an accumulation of U87 cells in sub-G1 and U138 cells blocked in G2/M associated with an increase in the p53 wt level after exposure to 3.3 µM 2-ME [67].

In conclusion, estrogens modulate three of the main cancer hallmarks—proliferation, migration/invasion and survival—that should be targeted for precision medicine.

#### 2.4.2. The Pro-Tumoral Role of Aromatase

Although the impact of estrogens on the progression of gliomas is still debated, the role of aromatase, the estrogen-producing enzyme was the subject of several studies. Based on 36 biopsies of astrocytomas from grade I to IV, Dueñas Jiménez and colleagues [68] concluded that the higher the grade the more aromatase was produced [68]. Since high level of aromatase was associated with a poor survival prognosis, treatment of T98G human GBM cells with vitamin D, dexamethasone and mifepristone (progesterone receptor (PR)/glucocorticoid receptor (GR) antagonist), which increased aromatase expression could trigger tumor malignancy [68,69]. Melatonin, which could have aromatase inhibitory properties, had opposite effects on C6 cells and led to a decrease in cell proliferation [70]. A concentration of 0.1 µM of nano-encapsulated letrozole, a competitive aromatase inhibitor, decreased the proliferation and migration on cells derived from patients GBM expressing aromatase [71]. Orthotopic C6 cell transplants in rats showed that a dose of 4 mg/kg letrozole resulted in a decrease in tumor volume by 75% after 8 days of treatment [72]. These different studies all tended to demonstrate that aromatase was a factor favorable for tumor progression.

In line with clinical data, exogenous estrogen supplementation could be beneficial against gliomagenesis whereas endogenous estrogen production via aromatase up-regulation stimulated tumor growth.

#### 2.4.3. Exogenous Progesterone Exposure: A Dose-Dependent Modulation of Malignancy

High concentrations of progesterone ranging from 20 to 80 µM caused a decrease in cell viability and increased the p53 protein level in U87, U87 dEGFR (genetically modified U87 cell line which stably expressed the EGFRvIII mutant form of epidermal growth factor receptor), U118MG cells (human male GBM cell line) [73,74]. The anti-proliferative effects of high doses of progesterone would be due to a slowdown of glycolytic metabolism in these cell lines [75]. Recently, Altinoz and colleagues [76] explored the proteomic changes in U87 and A172 human GBM cells treated by 100 µM or 300 µM progesterone: detoxification of reactive oxygen species, cellular response to stress, glucose metabolism, and immunity-related proteins were affected by these high doses of progesterone, leading to a slowdown of cell proliferation [76].

Conversely, low concentrations of progesterone ranging from 0 to 5 µM increased cell viability and proliferation [73]. Indeed, another study reported that 10 nM progesterone caused an increase in the mRNA level of PIBF (progesterone-induced blocking factor), a target gene for PR in U87 cells, associated with increased proliferation, migration and invasion of two human male GBM U87 and U251 cells [77]. Progesterone doses ranging from 1 nM to 10 µM were tested on human male U373 and human female D54 cell lines, respectively, from grade III and from grade IV tumors. Only the 10 nM dose caused a significant increase in the number of U373 cells with an increase in the number of S-phase cells (61% compared to vehicle). No significant effect was observed on D54 cells, which could be explained by a difference in the expression of PR isoforms between both cell lines [78]. Another study conducted by Piña-Medina and colleagues [79] indicated that 10 nM progesterone promoted migration and invasion of D54 cells [79].

In vivo, U87 xenografted nude mice exposed to 100 or 200 mg/kg of progesterone showed a 73.61% and 60.65% decrease in tumor volume, respectively, and a 60% survival gain for both conditions. Both doses of progesterone appeared to decrease the levels of vascular endothelial growth factor (VEGF) and matrix metallopeptidase (MMP) 9. Similarly, the levels of cleaved caspase-3, p53 and Bad increased and those of Bcl-2 decreased, suggesting a pro-apoptotic effect of progesterone [74]. In an orthotopic mouse model grafted with U87 cells, exposure to high doses of progesterone (100mg/kg) resulted in a 47% decrease in growth rate, a 45% reduction in tumor size and a 43% survival benefit compared to untreated mice. Notable effects were observed on the general well-being of the animals with increased travel and resting time after 4 weeks of treatment without deleterious effects on other peripheral tissues including liver and kidney. These beneficial effects of progesterone are believed to be associated with decreased cell proliferation, angiogenic ability and increased apoptosis. A similar study was performed on rat xenografted with U87 cells into the cerebral cortex and described an opposite effect for lower dose of progesterone (4mg/kg). The effect of progesterone, mediated through PR, would be responsible for an increase in tumor area and infiltration length [80]. In an in vivo model, U373 cells were implanted in the motor cortex of rat exposed to 1mg of progesterone. This resulted in increased tumor growth and infiltration in deeper structures of the brain compared to untreated animals. Colocalization of Ki-67 and sex-determining region Y-box (SOX2) was found in 63% of the cells after progesterone treatment, demonstrating its impact on proliferation and stemness [81].

Taken together, these studies demonstrated the impact of progesterone exposure on several hallmarks of gliomagenesis, though depending on the model and doses used. Therefore, dose response co-treatment of glioma cells with 17β-estradiol or/and progesterone might provide new insight that supports the clinical data reported above.

#### 2.4.4. Exogenous Androgen Exposure: High Levels and Increased Malignancy

High levels of circulating androgens were associated with increased glioma risk in men [82,83]. Bunevicius and colleagues [84] suggested a greater prenatal testosterone and lower prenatal estrogen exposure in brain tumor patients [84,85]. Moreover, testosterone (100 nM) and DHT (10 nM) exposed U87, U251 and D54 GBM cells displayed enhanced proliferation, migration and invasion [86,87]. In rats xenografted with GBM, experimental castration reduced the incidence of tumor development and increased the time between implantation and death [88].

DHEA levels also appeared to be present in greater amounts in the serum of GBM patients than in healthy controls and were thought to be associated with a decrease in tumor cell sensitivity to TMZ. A 5 µM dose of DHEA counteracted the effects of TMZ on DNA damage in the male astrocytoma A172 cell lines and in primary Pt#3 cells, isolated from the GBM tissue of a male patient [89]. The action of DHEA seemed to be mediated by Sp-1, a transcriptional factor that became phosphorylated via the induction of the Lyn/Akt pathway, deacethylated by histone deacetylase (HDAC) 1/2 and further associated with proliferating cell nuclear antigen (PCNA) to trigger DNA repair [89]. The effects became more complex when considering the impact of the different epimers of one hormone. The Δ5-androstene-3β, 17α-diol (17α-AED) inhibited T98G cell proliferation by more than 90% (IC50: 14.28 µM ± 5.25) while the 17β-epimer had no effect. The 17α-AED was believed to induce autophagy of human male GBM T98G, U87, LN-Z308 cells and primary GBM6 cells as evidenced by the increase in Beclin-1 autophagic marker and the conversion of LC3-I to LC3-II [90].

In conclusion, elevated levels of endogenously produced and exogenous androgens correlated with increased tumor malignancy and resistance to treatment in vitro and in vivo.

#### 2.4.5. Exogenous Glucocorticoids Exposure: Still Incomplete and Debated Data

Among exogenous glucocorticoids, dexamethasone is the most studied, because of its extended use in clinics although controversial benefits. Patients with high-grade glioma (III and IV) may develop vasogenic edema and increased intracranial pressure. To relieve the patient, corticosteroid therapy is recommended with the use of dexamethasone. Its use is still controversial as there are few clinical trials to determine its optimal dose, frequency of use and whether it is really beneficial to the patient in the treatment of high-grade gliomas [91,92,93]. A recent review published by Dubinski and colleagues [94] described the impact of dexamethasone on GBM progression [94]. Two hypotheses faced each other: some researchers attributed beneficial effects to dexamethasone, while others drew the opposite conclusions. In preclinical studies, the beneficial effects described were a decrease in proliferation, motility, migration, invasion and tumor vascularization. The negative effects cited were an increase in proliferation, angiogenesis, invasion and anti-apoptotic effects. In addition, dexamethasone in combination with TMZ would decrease the efficacy of TMZ. Although preclinical studies contradicted each other, clinical studies described dexamethasone as a risk factor for poor survival (12.7 vs. 22.6 months). A recent study indicated that treatment with cortisone for 24 h resulted in a decrease in the proliferation of C6 cells in a dose-dependent manner due to the translocation of GR in the nucleus. These effects were GR dependent since the addition of mifepristone, a GR antagonist blocked the effect of cortisone [95]. No clinical studies addressed the specific role of either GRα or GRβ isoforms or mineralocorticoid receptors that are both expressed in glioma cells, although this could help to understand the precise mechanism of action of dexamethasone or other corticoids.

Indeed, to establish precisely the role of steroid hormones in gliomagenesis, the relationship between the synthesis/exposure to hormones and the expression/functionality of their receptors must be investigated. For estrogens and progesterone in particular, the expression of membrane isoforms was not addressed despite their potential involvement in tumor progression through membrane non-genomic signaling pathways, as reported for numerous solid tumors.

## 3. Steroid Receptors and Downstream Signaling

Two types of receptors can mediate the effects of hormones: the membrane receptors and nuclear receptors. Nuclear receptors are mainly associated with the action of steroid hormones and other lipophilic messengers, such as vitamin D and retinoic acid, that are able to cross the plasma membrane by diffusion or facilitated transport. When activated by their ligand, these nuclear receptors act as transcription factors to directly regulate the expression of their target genes through the so-called genomic pathways [96]. Membrane receptors are more likely to be associated with the action of polypeptide hormones (growth hormone, insulin, etc.), neurotransmitters and growth factors (EGF, VEGF, insulin-like growth factor (IGF), etc.). The specific interaction of a ligand with its membrane receptor triggers a non-genomic signaling cascade involving second messengers and effectors that produce a rapid effect [97].

### 3.1. Estrogen Receptors, Complex and Interconnected Signaling Pathways

**Figure 2:**
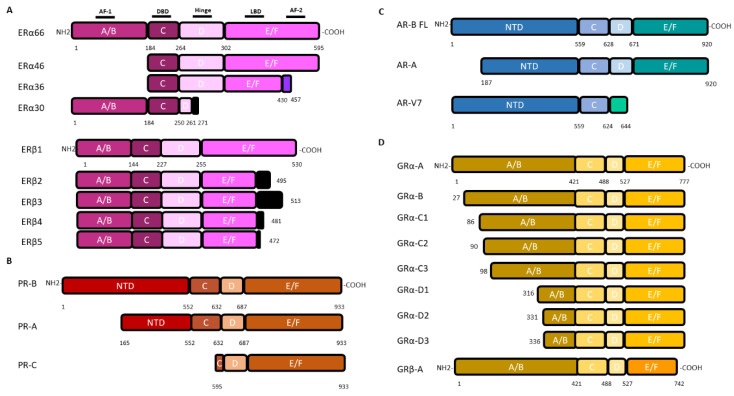
Nuclear steroid receptor isoforms (not drawn to scale). Nuclear receptors are characterized by their 6-domain structure. As shown for ERα from the NH2 to the COOH-terminal end, (i) the A/B domain, with the AF-1 region (Activation Function 1) which allows ligand-independent transactivation, (ii) the C domain with the DNA Binding Domain (DBD), (iii) the hinged D domain, (iv) the E/F domain which has the LBD (Ligand Binding Domain) and the AF-2 (Activation Function 2) region for ligand-dependent transactivation. (**A**): The canonical estrogen receptors ERα66 and ERβ1 are encoded by the *ESR1* and *ESR2* genes, respectively. The ERα variants, ERα46 and ERα36, lack the A/B domain. The ERα36 variant has a truncated E/F domain and a small specific C-terminal sequence of 27 amino acids (green box). The ERα30 variant retains only the A/B domain, the C domain and part of a D domain with a short specific sequence of 10 amino acids (black box). The 4 variants of ERβ differ in a truncated E/F domain and a small C-terminal sequence (black box). Adapted from [98]. (**B**): PR isoforms are encoded by the *PGR* gene. Two alternative promoters located upstream the coding sequence trigger the transcription of the full-length PR-B and the N-terminal truncated protein PR-A whereas another downstream promoter gives rise to the PR-C encoding transcript. (**C**): AR isoforms are encoded by the unique *AR* gene. Among the 23 isoforms, only the full-length one (AR-FL) and the AR-V7 variant, which lack the hinge and E/F domains but have a short C-terminal specific sequence, are mentioned because of their involvement in gliomagenesis. Adapted from [99]. (**D**): GR isoforms are encoded by the GR gene through alternative splicing of exons 9α/9β, giving rise to GRα or GRβ proteins, respectively, or use of different translational initiation sites that produce multiple GRα isoforms termed A through D (A, B, C1-C3 and D1-D3). Since GRα and GRβ share a common mRNA domain that contains the same translation initiation sites, the GRβ variant mRNA may also produce the B-D isoforms, although not yet biologically demonstrated.

Estrogen effects are mediated by nuclear estrogen receptors, ERα (encoded by the *ESR1* gene) or ERβ (encoded by *ESR2*) that can translocate into the nucleus and modify directly the expression of their target genes, as well as by the membrane forms of ERα and ERβ, or the transmembrane receptor GPER (g protein coupled estrogen receptor 1), that trigger non-genomic estrogen signaling. The *ESR1* and *ESR2* genes, respectively, encode 4 (ERα66, 46, 36, 30) and 5 (ERβ1-5) isoforms, through alternative promoters and splicers (Figure 2).

Although GPER has been detected in the hippocampus and is believed to be responsible for activation of the MAPK/ERK pathway to promote synaptic plasticity in normal brain, no information is available on its cellular location [100,101]. In addition, no data are currently available on its role in gliomagenesis.

The expression level of ERα and ERβ varies between healthy and tumor brain tissues but also according to the grade of the tumor. In normal brain, ERα is found mainly in the ventromedial hypothalamic nucleus, subfornical organ and in arcuate nucleus while ERβ is found in neurons and astrocytes of the olfactory bulb, cerebellum, paraventricular and hippocampus. This distribution into the different regions of the brain implies that each type of receptor may participate in specific neural/glial functions [102]. Recently, many isoforms of ERα and ERβ have been detected in normal brain and astrocytoma: the detailed cartography of these receptor localization as well as their expression level, distinctive mode of action and effect remain to be elucidated.

**Figure 3:**
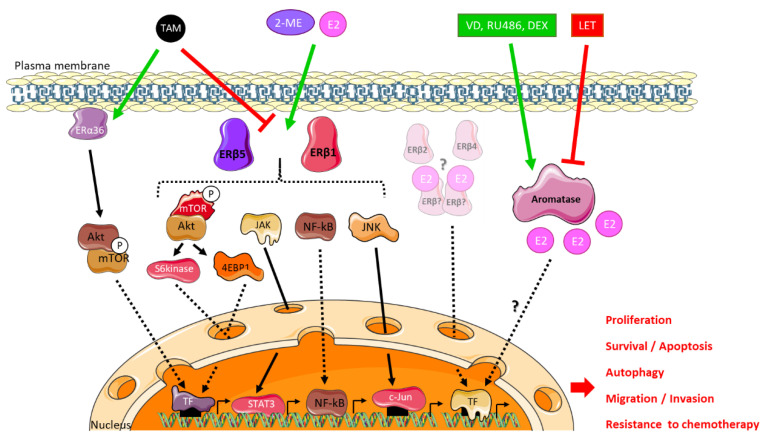
Estrogen and anti-estrogen signaling in glioblastoma. Glioblastoma cells are sensitive to exogenous 17β-estradiol (E2) and 2-methoxyestradiol (2-ME) and the anti-estrogen tamoxifen (TAM) exposure through ERβ1, 2, 5 and ERα36 receptors that trigger non-genomic signaling. Tamoxifen-induced ERα36-dependent pathway involves PI3K-Akt and mTOR signaling to enhance autophagy and resistance to anti-cancer therapy. In the presence of 17β-estradiol or 2-methoxyestradiol, ERβ1 and ERβ5 nuclear receptors stimulate mTOR, JAK/STAT, NF-kB or JNK signaling to impact cancer cell proliferation, migration/invasion, survival or resistance to chemotherapy or may directly modulate the expression of their target genes. ERβ2 isoform is expressed in most glioblastoma cell lines or patient tumor samples but the downstream signaling remains to be determined. Endogenous production of estrogens by aromatase may be stimulated by an enhanced expression of the enzyme through vitamin D (VD), mifepristone (RU486) or dexamethasone (DEX) exposure, leading to increased tumor cell proliferation whereas the administration of letrozole (LET), a competitive inhibitor of aromatase activity, results in tumor volume reduction.

An immunohistochemical study of low- and high-grade astrocytomas sought to characterize the expression of ERα in these two groups using the NCL-ER-6F11 ERα antibody. The expression of ERα correlated with the grade of the tumor: unlike the low grades, the high grades no longer expressed ERα [103]. Conversely, two publications recently reported the high expression ERα36 in U87 and U251 GBM cell lines [104,105]. ERα36 was found anchored to the cytoplasmic membrane of these cells via Caveolin-1. Furthermore, its expression was stimulated by long-term tamoxifen (TAM) exposure, leading to subsequent inhibition of TAM-induced cell cycle arrest and apoptosis as well as enhanced autophagy through the modulation of Akt/mTOR pathway [105] (Figure 3). ERα36 knockdown in these TAM-resistant cells restored TAM sensitivity.

As for ERα, an immunohistochemical study confirmed that high-grade tumors expressed less both ERα and ERβ [106]. Conversely, Liu and colleagues [106] used a pan-ERβ antibody to show that this receptor was more expressed in tumoral tissue compared to healthy one even if its expression varies from one patient to another [106]. Those who expressed a high level of ERβ seemed to have a better survival than those who expressed a low level [107].

Since the role of ERβ remained unclear, agonists such as LY500307 and Toosendanin were tested. These two molecules increased the expression of ERβ, promoted apoptosis and decreased cell proliferation. In mice implanted intracranially in the right striatum with U251 cells, a dose of 5 mg/kg/day of LY500307 for 28 days reduced tumor volume [108]. Similarly, the treatment (1 mg/kg of Toosendanin) of athymic nude mice xenografted with U87 cells decreased the size of the tumor by approximately 2-fold. The effect of Toosendanin was mediated by an increase in p53 expression [109].

Although ERβ appeared to play an overall anti-tumoral role, estrogenic signaling is more complex and involved the expression/interaction/functionality of numerous isoforms. In C6 and F98 rat cells, ERα/ERβ balance modulated estrogen-dependent expression of Cx43, a gap junction protein strongly expressed in low grade but weakly expressed by high grade astrocytomas [65,110]. Further studies dedicated to unique isoforms are required to associate a function to each isoform alone or in combination. Recently, Liu and colleagues [111] explored the repertoire of ERβ isoforms in GBM tumors and cell lines [111]. They showed that GBM cells predominantly expressed ERβ1 and ERβ5, along with low levels of ERβ2 and ERβ4.

#### 3.1.1. Focus on ERβ5, an Ambivalent Receptor

ERβ5 seemed to be the main form of ERβ in human gliomas, its expression being even induced in pathological conditions, resulting in higher levels in GBM than in low-grade astrocytoma [111,112]. In order to study the role of ERβ5 in U87 and U251 GBM cells, Liu and colleagues [111] generated knockout (KO) cell lines for all ERβ isoforms by CRISPR/Cas9 and then complemented by ERβ5 expression vectors into the same cells [111]. ERβ5 enhanced migration/invasion through Akt/mTOR/4EBP1 (ErbB3-binding protein 1) pathway (Figure 3). In vivo, ERβ KO mice grafted with ERβ5-expressing cells displayed no survival difference compared to ERβ KO [111]. Another study showed that ERβ5 could be associated with a decrease in cell proliferation and invasive potential through reduced MMP2 activity [112]. These contradictory data may result from ERβ5 interaction with multiple partners, depending on the cellular context and treatment. Up to now, no data are available in other CNS tumors.

#### 3.1.2. Focus on ERβ1, a Tumor Suppressor?

Liu and colleagues [111] carried out the same approach to study the role of ERβ1 [111]. U87 and U251 GBM cells expressing ERβ1 only triggered a decrease in cell viability through NF-kB downregulation, JAK/STAT and mTOR/S6kinase signaling and a lower migratory and invasive potential, due to a decrease in MMP2 activity [111,112] (Figure 3). The anti-tumor role of ERβ1 was confirmed in an in vivo model in which the mice grafted with ERβ1 expressing cell had a better survival rate than KO control ones [111].

Zhou and colleagues [113] explored the role of ERβ1 in GBM cells [113]. Using RNA-sequencing, they compared the transcriptome of empty vector and ERβ1 overexpressing U87 cells. ERβ1 overexpression down-regulated the expression of genes linked to DNA damage checkpoint regulation, DNA damage response, DNA repair, ATM signaling pathways and upregulated those of cell cycle. ERβ1 overexpression was also shown to potentiate TMZ and other chemotherapeutic agent treatment by promoting apoptosis and the cell cycle arrest in vitro and survival of in vivo [113].

Collectively, these results indicated that ERβ isoforms differentially modulate the mTOR, NFkB and JAK–STAT pathways in GBM cells, and suggested that ERβ1, but not ERβ5, displayed tumor-suppressing functions in GBMs.

### 3.2. Progesterone Receptors: The PR-A Isoform

Progesterone is mainly secreted by the cells of the corpus luteum of the ovaries and the placenta. It is involved in the control of embryogenesis, menstrual cycle and pregnancy. Progesterone exerts its function by interacting with its intracellular PR. PR is expressed as two major isoforms, PR-A and PR-B, but also as PR-C from alternative promoter use. PR-B is the canonical form of PR (933 amino acids) and PR-A and PR-C are N-terminus truncated isoforms (769 and 358 amino acids, respectively) (Figure 2). These isoforms have a different three-dimensional structure and therefore interact with various partners, which drive the cellular response. The nuclear forms of PR-A and PR-B are able to trigger genomic signaling, bind specific progesterone response elements (PRE) and activate progesterone target genes. PR-B is also capable of inducing non-genomic pathways initiated at the cell membrane by cell surface PRs, ion channels or cytoplasmic second messenger cascades [114,115]. PR-C retains the progesterone binding but not the DNA binding domain (Figure 2). In brain, membrane PRs are found in the forebrain, the hypothalamus and the hippocampus and trigger ERK, cAMP/PKA, PKG signaling, Ca^2+^ influx/PKC activation, PI3K/Akt pathway [46,116]. These different mechanisms are involved in particular in the regulation of intracellular calcium, proliferation and cell viability [117].

Most studies looking at PR in astrocytomas used two cell models: U373 cells originating from grade III and D54 from grade IV tumors. Both cell lines expressed PR-A and PR-B. Estrogen (10 nM) increased PR-A and PR-B levels, and progesterone (10 nM) increased cyclin D1 levels in U373 cells [118,119]. These results suggested that progesterone signaling could depend on the grade of human astrocytoma cells and crosstalked with estrogen response.

As with ERs, it is possible that each of the PR isoforms has specific functions. Despite very few studies specific to each isoform being carried out, it appeared that in high-grade astrocytomas (III and IV), PR-B is the main isoform. All grade IV but only 83% of grade III tumors expressed PR-B [120]. Treatment with progesterone on U373 cells overexpressing PR-A tended to decrease cell proliferation [118]. This effect seemed to be confirmed on patient astrocytic tumors with a negative correlation between the level of Ki-67 and PR-A [121]. No PR-C specific data have yet been published.

### 3.3. Androgen Receptors

Androgens and androgen receptors (AR) contribute to the development and maintenance of the male sexual phenotype. Two isoforms of AR were initially discovered (Figure 2): AR-A and AR-B. AR-B was the canonical sequence of AR (920 amino acids) whereas AR-A was lacking the first 187 amino acids (733 amino acids). Then, over 20 AR variants have been identified, among which AR-V7 has been shown to be the most abundant of the AR splice variants [122]. In the brain, ARs are expressed in neurons and in the glia of the medial amygdala, the preoptic area, the hypothalamus, the cerebellum and the dentate gyrus of the hippocampus [46].

Similar to the other nuclear receptors, there are genomic and non-genomic AR-dependent signaling pathways. Once activated by their ligands, the ARs are able to bind androgen response elements (AREs) which trigger transcription of the AR target genes. The non-genomic pathway may be activated by nuclear ARs described above, recently described transmembrane ARs (mARs) GPRC6A (g protein-coupled receptor family C group 6 member A) and ZIP9 (zinc transporter member 9), or by changes in membrane fluidity [123,124].

The first immunohistochemical analysis of low-grade and high-grade gliomas showed that AR protein expression level neither depended on tumor grade nor was a reliable indication for patient survival [125]. Recent studies seemed to provide conflicting data. Bao and colleagues [126] indicated that high grade expressed more AR than low-grade tumors [126]. Liu and colleagues [106] published the opposite results with high grades expressing less AR [106]. These divergent results make it difficult to interpret the role of AR in tumor progression. A single study has shown by Western blot that 30% of GBMs expressed the AR-V7 isoform [127].

From a functional point of view, AR seemed to promote tumor growth in glioma: an increase in cell death and an accumulation of cells in sub-G1 are observed when blocking the AR activity by specific inhibitors (bicalutamide or enzalutamide) or protein expression by siRNA in A172, U87 and T98G cells. Moreover, mice xenografted with U87 cells and treated with a dose of 20 mg/kg of enzalutamide showed a 72% decrease in tumor mass [127]. Several mechanisms were proposed to explain the oncogenic role of AR. In T98G cells, a decrease in ARA54, which is an AR co-activator, was associated with a decrease in proliferation and an accumulation of cells in G1 phase [128]. In U87 cells, inhibition of VCP/P97-interacting protein (SVIP) by AR led to an increase in proliferation and a decrease in p53 expression [126]. In the same cells, AR could bind to SMAD3 and block its activity and therefore antagonized transformation growth factor (TGFβ) signaling, thus promoting cell growth and avoiding apoptosis [82]. In low-grade gliomas, the expression of a long non-coding RNA, called DRAIC (for downregulated RNA in cancer, inhibitor of cell invasion and migration), has been shown to be associated with better patient survival. AR could bind to the DRAIC for locus and repress its expression, thereby promoting cell migration and invasion [129].

In conclusion, it seems that AR has pro-tumor properties by promoting proliferation, migration and invasion of tumor cells. In-depth studies on the different isoforms of AR are needed to decipher their respective mechanisms of action; in particular, AR-V7 expressed in 30% of GBMs that may be used as a tumor marker.

### 3.4. Glucocorticoid Receptors: Multiple Isoforms but Limited Data

The effects of glucocorticoids are mediated by nuclear glucocorticoid type I (i.e., mineralocorticoid receptors (MRs)) and type II (i.e., glucocorticoid receptors (GRs)) receptors. There are two GR isoforms: GRα and GRβ both located in the cytoplasm. The GRβ isoform (742 amino acid) results from alternative splicing of exon 9 at the C-terminal region. GRβ has no glucocorticoid-binding domain and is believed to act as a dominant negative inhibitor of GRα. When glucocorticoids bind to the canonical form GRα (777 amino acids), the complex translocates into the nucleus where it can bind to DNA. There are also seven isoforms of GRα and GRβ resulting from leaky scanning and ribosomal shunting that differs from the N-terminal region and could induce activation or repression of various genes (Figure 2).

GR is expressed in neurons, oligodendrocytes, astrocytes and microglia of the brain and spinal cord [130,131]. Very little information is available on the location of each isoform in the human brain, but the GRβ isoform is much less expressed than GRα in the hippocampus (ratio 14,500:1) [132].

Few studies on the expression of GR isoforms in astrocytoma patients have been published so far. A study of 12 GBM samples showed that they all expressed GR. The concentration of GR seemed to be higher at the periphery of the tumor compared to the center and even lower in normal brain tissue [133]. Similar studies showed different results with some tumors that did not express GR [133,134]. However, the pre-operative glucocorticoid intake was not taken into account, although it could reduce GR expression.

The regulation of the level of GR in gliomas remains poorly documented. The impact of glucose deprivation and hypoxic condition on the expression of GR was addressed in U87 cells. Only hypoxia increased the rate of GR [135,136]. Inhibition of the activity of IRE1/ERN1 (inositol requiring the enzyme 1/endoplasmic reticulum at signaling of the nucleus 1) was also responsible for an increase in the level of GR although it limited the effect of hypoxia [135].

Functional in vitro studies tended to show that the GRβ isoform could be involved in the progression of gliomas by participating in cell proliferation and migration. Knock down of GRβ in U118 glioma cell line resulted in a cell growth inhibition [137]. These data appeared consistent since cortisone would activate only the GRα isoform, described as a repressor of the GRβ pro-tumoral activity [137,138].

In conclusion, the role of GR appears to be isoform specific with GRβ described as pro-tumoral. In patients, GR is expressed in greater amounts at the periphery of the tumor, possibly participating in tumor expansion. Knowing precisely which GR isoform is present in the periphery could be used as a marker of tumor aggressiveness.

## 4. Conclusions and Future Prospects

Glioma is, in adults, the most common brain tumor, very aggressive with a very poor prognosis. Epidemiological data show that men are more affected than women, raising the potential role of steroid hormones in gliomagenesis. Most of the studies presented in this review, from the oldest to the most recent, bring convergent findings and make it possible to conclude that gliomas and, more particularly, astrocytomas, are indeed hormone-sensitive tumors, even if the molecular subtypes currently in use at diagnosis do not account for steroid biosynthesis enzymes or receptors. To further unravel the impact of estrogens, progestogens and androgens on gliomagenesis, some teams have focused on the molecular mechanisms involved in their response pathways. However, the discovery of numerous membrane and nuclear steroid receptor isoforms and, in particular, estrogen receptors, makes this mechanistic more complex. It is now essential to characterize more precisely the role of each isoform, and the crosstalks between hormone responses to better understand the signaling pathways and characterize downstream targets impacted by steroids in astrocytomas. Ultimately, these data will contribute to a better understanding of these very poor prognosis tumors and open up new therapeutic strategies for precision medicine.

## 5. Data Source Extraction and Management

We conducted a systematic search of the literature in PubMed and Google Scholar databases, between 1st March 2020 and 1st October 2020. The following key terms were searched in the title, abstract or keywords of the published papers: “glioma or astrocytoma or glioblastoma” and “classification”, “treatment”, “incidence”, “gender or sex”, “contraceptive pills or hormone replacement therapy”, “pregnancy”, “aromatase expression or activity”, “estrogen or estradiol or estrogen receptor”, “androgen or testosterone or dihydrotestosterone or androgen receptor”, “progesterone or progesterone receptor”, “cortisol or corticosterol or glucocorticoid receptor”. It should be noted that (i) most publications cited in this review do not distinguish between glioma, astrocytoma or oligodendroglioma, and consequently are referred to as “glioma” in the present review; (ii) the detailed reference of a sample or cell line to a particular subtype defined in the 2016 classification is usually not reported by the authors and therefore not mentioned in this review; (iii) the vast majority of studies that address the impact of steroid hormones or receptors on glioma use IDH1 wild-type GBM cell lines for in vitro studies or for animal xenografts. Therefore, we mentioned the IDH1 status only for the mutated cell lines.

## Figures and Tables

**Table 1:** WHO 2007 classification of malignant gliomas (adapted from [1,2]).

Histologic Class		Grade	50% Overall Survival (years)	Mean Age at Diagnosis
**Low-grade glioma**	Astrocytoma	II	4–10	42
	Oligodendroglioma	II	8–20	43
	Oligoastrocytoma	II	5–12	44
**High-grade glioma**	Anaplastic astrocytoma	III	2–5	57
	Anaplastic oligodendroglioma	III	2–10	61
	Anaplastic oligoastrocytoma	III	2–8	52
**High-grade glioma**	Glioblastoma	IV	1–2	45–75

**Table 2:** WHO 2016 classification of malignant gliomas (adapted from [3,4]).

Histologic Class	Molecular Subtype			Grade	50% Overall Survival (years)	Mean Age at Diagnosis
	IDH1	1p/19q	Other Genetic Alteration (Not Required for Diagnosis)			
**Oligodendroglioma**	mut	codel	TERT mut	II/III	>15/10	35–45
**Diffuse astrocytoma**	mut	wt	ATRX mut, p53 mut	II/III	11/9	35–45
**Diffuse astrocytoma**	wt	wt	p53 mut, PTEN mut, PIK3, EGFR amplification, CDKN2A/B deletion, CDK4, BRAF, ATRX mut	II/III	5/2–3	45–50
**Glioblastoma**	mut	wt	p53 mut	IV	2.5	50–60
	wt	wt	PTEN mut, EGFR amplification	IV	1.5	50–60
